# Refining Reproductive Parameters for Modelling Sustainability and Extinction in Hunted Primate Populations in the Amazon

**DOI:** 10.1371/journal.pone.0093625

**Published:** 2014-04-08

**Authors:** Mark Bowler, Matt Anderson, Daniel Montes, Pedro Pérez, Pedro Mayor

**Affiliations:** 1 San Diego Zoo Global Institute for Conservation Research, Escondido, California, United States of America; 2 School of Psychology, University of St Andrews, St. Andrews, Fife, Scotland; 3 Yavari: Conservación y Uso Sostenible (YAVACUS), Iquitos, Loreto, Perú; 4 Departament de Sanitat i Anatomia Animals, Universitat Autònoma de Barcelona, Bellaterra, Spain; University of Western Ontario, Canada

## Abstract

Primates are frequently hunted in Amazonia. Assessing the sustainability of hunting is essential to conservation planning. The most-used sustainability model, the ‘Production Model’, and more recent spatial models, rely on basic reproductive parameters for accuracy. These parameters are often crudely estimated. To date, parameters used for the Amazon’s most-hunted primate, the woolly monkey (*Lagothrix* spp.), come from captive populations in the 1960s, when captive births were rare. Furthermore, woolly monkeys have since been split into five species. We provide reproductive parameters calculated by examining the reproductive organs of female Poeppig’s woolly monkeys (*Lagothrix poeppigii*), collected by hunters as part of their normal subsistence activity. Production was 0.48–0.54 young per female per year, and an interbirth interval of 22.3 to 25.2 months, similar to parameters from captive populations. However, breeding was seasonal, which imposes limits on the maximum reproductive rate attainable. We recommend the use of spatial models over the Production Model, since they are less sensitive to error in estimated reproductive rates. Further refinements to reproductive parameters are needed for most primate taxa. Methods like ours verify the suitability of captive reproductive rates for sustainability analysis and population modelling for populations under differing conditions of hunting pressure and seasonality. Without such research, population modelling is based largely on guesswork.

## Introduction

In the Amazon region, wildlife subsistence hunting is a traditional source of food for rural human populations [1]. Woolly monkeys (*Lagothrix* spp.) are large-bodied Ateline primates weighing around 6–10 kg, with males weighing around 2 kg more than females [6], [7]. Consequently, they are the most frequently hunted primate in Amazonia, representing an important source of meat in the region [2]–[6]. For this reason, several studies have examined the susceptibility of woolly monkeys to hunting [8]–[12].

Like many primate taxa, woolly monkeys have recently been subject to taxonomic revision. They were previously described as a single species, *Lagothrix lagothricha,* split into four subspecies; *L. lagothricha lagotricha, L. lagothricha cana, L. lagothricha poeppigii* and *L. lagothricha lugens* [13]. However, these have more recently been given full species status [14]–[16], a classification that has become widely used [6], [17]. Additionally, the yellow-tailed woolly monkey, previously *Oreonax flavicauda* [15], [16], [18], is now considered to be a fifth species of *Lagothrix* [19], [20]. The genus now therefore contains Vulnerable (*L. lagotricha* and *L. poeppigii*), Endangered (*L. cana*) and Critically Endangered (*L. lugens* and *L. flavicauda*) species [17].

The conservation status of species and the implementation of *in situ* and *ex situ* conservation programs are often guided by assessments of the vulnerability to extinction or sustainability of hunting of the target species in a given area [17]. A range of models have been used to examine the vulnerability of primates to hunting, many of which use measures of their reproductive performance to estimate key parameters (Table 1). One of the most-used is the ‘Production Model’ [8], which has become a standard model in sustainability analyses [10], [21]–[25]. A key parameter of the Production Model is the *intrinsic rate of natural increase* (*r_max_*), estimated using Cole’s Equation [26]:

**Table 1:** Models using reproductive parameters of *Lagothrix* to assess the sustainability of hunting on the species.

Model	Species of the population being modelled [15], [16]	Basic reproductive parameters used and sources
Abundance, density, or standing biomass comparisons	*Lagothrix poeppigii* [65]	None
	*Lagothrix poeppigii* [67]	None
	*Lagothrix poeppigii* [68]	None
	*Lagothrix poeppigii* and *Lagothrix cana* [69]	None
	*Lagothrix* spp. [70]	None
	*Lagothrix poeppigii* [71]	None
	*Lagothrix lagotricha* [64]	None
Production model [8]	*Lagothrix poeppigii* [72]	*a*, *w* & *b* [8] * ^1^
	*Lagothrix cana* [23]	*a*, *w* & *b* [8] * ^1^
	*Lagothrix* spp. [73]	*a*, *w* & *b* [8] * ^1^
	*Lagothrix cana* [10]	*a*, *w* & *b* [8] * ^1^
Harvest model [80]	*Lagothrix poeppigii* [74]	*b* [8] * ^2^
	*Lagothrix poeppigii* [75]	*b* [8] * ^2^
Production model with survival probabilities [55]	*Lagothrix poeppigii* [55]	*a*, *w* & *b* [8] * ^1^and several alternative estimates of mortality cited within [55]
Stock recruitment model [81]	*Lagothrix poeppigii* [76]	none
Unified harvest model [82]	*Lagothrix poeppigii* [77]	*b* [8] * ^2^
Source sink models [83]	*Lagothrix poeppigii* [52]	*a*, *w* & *b* [8] * ^1^
Spatial models [11], [31]	*Lagothrix poeppigii* [31]	*a*, *w* & *b* [8] * ^1^
	*Lagothrix cana* [32]	*a*, *w* & *b* [8] * ^1^
Catch per unit effort [84]	*Lagothrix poeppigii* [78]	none
	*Lagothrix poeppigii* [79]	none

*a* is the age at first reproduction, *w* is the age at last reproduction, and *b* is the number of female off- spring per adult female per time unit.

*^1^ [8] used r_max_ for *Lagothrix* (0.14) from captive birth intervals [85] using estimates for *a*, *w* & *b* that can be traced back to [27] via [28] and [29], but see section 1.

**Figure 1:**
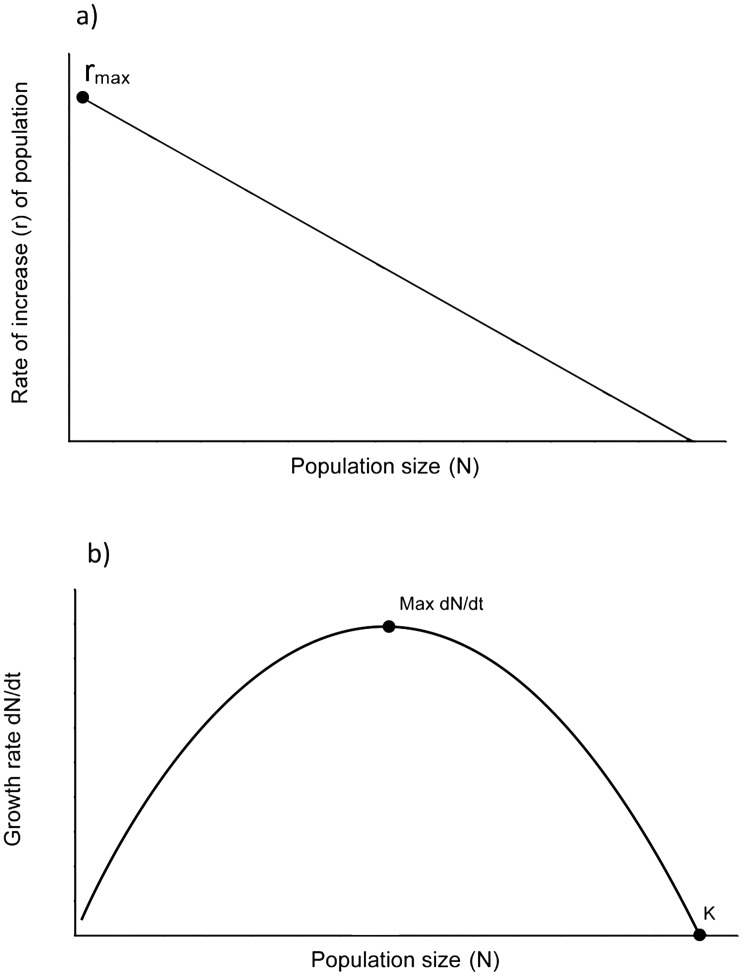
Logistic growth in density dependent populations. K = carrying capacity. Fig. 1a shows a linear decrease of population growth rate as population size increases and availability of resources per individual therefore declines. Fig. 1b shows an inverse u-shaped curve; population growth rates increase as the number of reproducing animals increases, until growth rates are resource limited in larger populations.

*^2^ *b* of 0.5 comes from [8] and citations within.



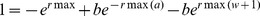
Where *a* is the age at first reproduction, *w* is the age at last reproduction, and *b* is the annual birth rate of female offspring. *r_max_* is important because when population growth is logistic (Figure 1), *r_max_* determines the initial growth rate of population as well as the maximum sustainable yield (MSY) of a hunted population.

For woolly monkeys, Robinson & Redford [8] use a value for *r_max_* of 0.14 calculated using the age at first (5 years) and last (20 years) reproduction and the inter-birth period (24 months) of captive populations to estimate the birth rate of a population not restricted by density dependent factors. The source of these parameters is a report on a captive population of *Lagothrix sp.* [27] cited in Wolfe al al. [28] and Robinson & Janson [29] that only recorded a total two births, one each to two different females, so an estimation of birth interval is not possible. To our knowledge, the only published estimate of captive woolly monkey birth intervals at the time came from a single estimated interval, between the birth of an infant that survived and a subsequent early abortion that was extrapolated to an estimated full-term [30], thus estimating an inter-birth period of 1.5 to 2 years that is possibly the actual source for Robinson & Redford [8]. All subsequent estimates for the sustainability of hunting of *Lagothrix* that use models requiring reproductive parameters, including more recent spatial models [11], [31]–[32], appear to have used these same estimates (Table 1), despite the availability of more up-to-date parameters.

Reproductive parameters from captive data are still limited, but estimates from larger samples now exist [33]. Wild-caught captive females (n = 36) first reproduce at 9 years of age and have a mean birth interval of 30 months, whilst captive-born females (n = 40) first reproduce at 6 years of age and have a mean interbirth interval of 25 months [33]. Woolly monkey females have a 21-day ovarian cycle with an estrus period of 1 to 8 days [27], [30], and gestation lasts approximately 225 days [27], [30], [33]. Normal litter size in the wild is one [34], [35], but in captivity births of three young have been observed [33]. Whilst there are better-supported estimates for the reproductive parameters of captive *Lagothrix* [33] than those typically used to date, captive woolly monkey populations are made up of individuals categorized as *Lagothrix lagotricha* before it was split into several species. The origin of these animals, and their current classification, is not typically recorded. Indeed breeding groups may have contained several taxa and hybrid animals. This situation is mirrored for populations of many other primate taxa held in captivity. Species-specific parameters should be sought for future models.

Di Fiore et al. [6] provide a calculation of r_max_ (0.16) based on more recent data, although again for an unspecified species of *Lagothrix*. These differences in *r_max_* are not trivial to calculations of sustainability. When growth is logistic, the MSY is rK/4 and scales linearly with ‘r’ [36]. So if *r_max_* is 0.16 [6] rather than 0.14 [29], then this 14% increase in *r_max_* is translated into a 14% increase in the potentially sustainable harvest. Levi et al’s [11] spatial model on the other hand appears less sensitive to variation in estimates of *r_max_*, predicting ‘extinction envelopes’, the area around community in which a hunted species does not occur, for *Lagothrix* of 6.7 km and 6.4 km (<5% difference) for *r_max_* of 0.14 and 0.16 respectively, for a single community hunting using guns in Manu NP, Peru.

We calculate wild reproductive rates for a hunted population of *Lagothrix poeppigii* in the North-eastern Peruvian Amazon to determine which of the available calculations of *r_max_* are most appropriate for use in sustainability studies and extinction modelling for this species and discuss important considerations for the refinement and use of primate reproductive parameters in modelling population change in response to hunting.

## Materials and Methods

Animals were collected on the Yavarí-Mirí River, from an area of 322,500 ha of continuous, predominantly non-flooding *terra firme* forest. The climate is typically equatorial with an annual temperature of 22°–36°C, a relative humidity of 80% to 100%, and an annual rainfall of 1500 to 3000 mm.

**Figure 2:**
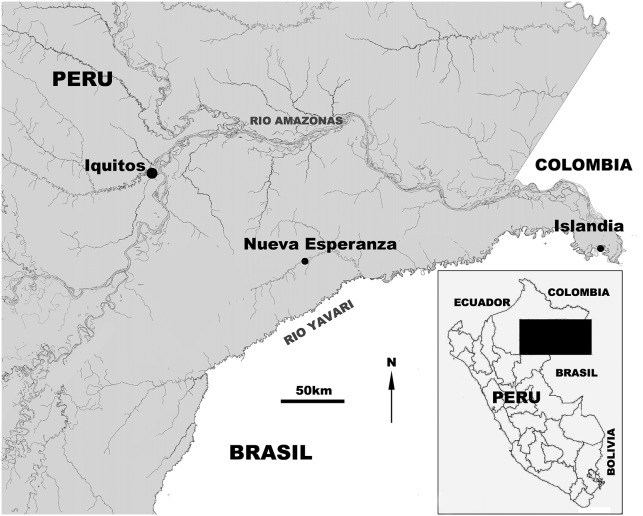
The Yavari Mirin River and surrounding area, including Esperanza, the main community in the area.

From 2004 to 2011, indigenous Yagua hunters living in or near the community of Esperanza (Figure 2), collected genital organs from 84 adult *Lagothrix poeppigii* females, as part of an ongoing participatory conservation program that involves local hunters in implementing community-based wildlife management [37], [38]. This assured that no animals were killed other than those harvested as part of local hunter’s normal activities. Indigenous people can hunt primates legally without a permit in Peru. The research was approved by the ‘Research Ethics Committee for Experimentation in Wildlife’ at the ‘Dirección General de Flora y Fauna Silvestre’ in Peru (0229-2011-DGFFS-DGEFFS). No animals were killed specifically for the research and hunters were not paid for the sample collection.

### Macroscopic Analysis

**Figure 3:**
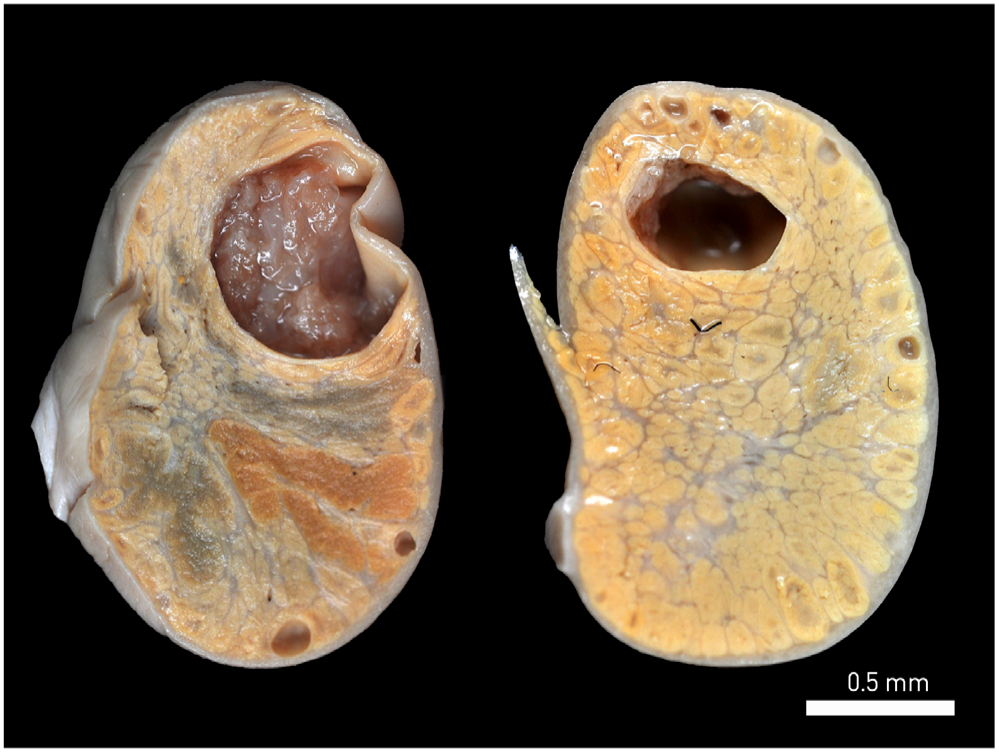
Active true corpora lutea (left) and a preovulatory antral follicle (right).

We maintained the genital organs of adult females in buffered 4% formaldehyde solution (v/v), and examined them for evidence of embryos or fetuses. We considered females with at least one embryo or fetus to be pregnant, defining the pregnancy stage as embryonic or fetal, using the reproductive characteristics of woolly monkey ovaries described in Mayor et al.[39]. We described non-pregnant females with ovaries containing active true corpora lutea (CL) as being in the luteal phase of the estrous cycle, while we considered females with ovaries bearing large antral follicles and lacking true CL to be in the follicular phase of the estrous cycle (Figure 3). In the absence of either large antral follicles or CL, we considered the ovaries inactive.

Based on the number of true CL, we determined the ovulation rate, expressed as the number of CL per female with ovulations. We determined fertilization rate as the total number of embryos or fetuses divided by the number of CL in pregnant females, the rate of ovum or embryo mortality as the difference between the number of CL and the observed embryos or fetuses, and the average litter size as the total number of embryos or fetuses per pregnant female. We recorded the fetal sex of each pregnancy.

We determined monthly conception dates by back-dating embryos or fetuses from the estimated age on the date when each female was collected, using a gestation length of 225 days [27], [33]. Since there is no characterization of fetal development in the Poeppig’s woolly monkey, we determined the embryo/fetal age primarily using a description of human fetal development [40].

### Reproductive Performance

We estimated reproductive performance following Mayor et al. [41]:

Ovulation rate = Number of CL/ovulating female.

Reproductive wastage = number of CL–number of embryos or fetuses in pregnant females.

Pregnancy rate = number of pregnant females/total adult females.

Pregnancy-days per year = 365 days/year*pregnancy rate.

Number of births per female per year = yearly pregnancy-days/gestation length.

Interbirth interval = gestation length/pregnancy rate.

Parturition-conception interval = interbirth interval-gestation length.

Yearly reproductive production = number of births per female per year*litter size.

Gross productivity = number of embryos or fetuses/number of adult females.

Gross fecundity = number of female fetuses/number of adult females.

### Statistical Analysis

To test the seasonality of reproduction, we transformed the estimated date of each parturition into the degrees of a circle (1^st^ January = 0.986° though to 31^st^ December = 360°) and applied circular statistics using a Raleigh’s Uniformity test using ‘R’ version 2.15.1 [42] and ‘R’ package ‘circular’ [43] to test whether parturitions were randomly distributed through the year (following [44]). The relative vulnerability to hunting of females was estimated by comparing the number of females in hunters’ registers with males and compared using a chi-square test using GraphPad Instat (version 3.01 for Windows 95, GraphPad Software Inc., San Diego, CA, USA: www.graphpad.com). Differences with a probability value of 0.05 or lower were considered significant.

## Results

There was no significant difference between the numbers of males and females hunted (89 males and 84 females; Yates’ chi-square, 0.092, P = 0.76).

Of the 84 sampled adult females, 60 (71.4%) were non-pregnant and 24 (28.6%) were pregnant females at different stages of pregnancy (Table 2). Non-pregnant females were classified as in follicular (n = 27; 45.0%) or luteal phases (n = 33; 55.0%). Two pregnant females were considered to be at the embryonic stage of pregnancy, with an embryo between 0.5 and 1 cm in size and with limb buds present. Twenty pregnant females were considered to be at the fetal stage of pregnancy, with developed eyelids, fingers and external genitalia, and all the vital organs in place. Due to the difficulty of diagnosing pregnancy during the 2 first weeks, we considered a possible underestimation of 10% of pregnancies in non-pregnant females in the luteal phase. Consequently, considering that 3.3 females in the luteal phase could be pregnant females in the earliest stage of pregnancy, the pregnancy rate could be as high as 32.5% (27.3 pregnant females).

**Table 2:** Reproductive performance of wild woolly monkeys (*Lagothrix poeppigii*) (n = 84) on the Yavarí-Mirí River.

Reproductive parameters	Total sample
Number of females	84
Number of non-pregnant females	60
Number of pregnant females	24
Number of foetuses	24
Pregnancy rate (%)	28.6–32.5
Pregnant days/year (days)	104–119
Non-pregnant days/year (days)	246–261
Parturitions/year/female	0.46–0.53
Interbirth interval (days)	692–787
Parturition-conception interval (days)	467–562
Litter size (young/parturition)	1.00
Foetal sexual ratio (F/M)	12/12
Yearly Reproductive Productivity (young/year/pregnant female)	0.46–0.53
Gross productivity (young/adult female)	0.286
Gross fecundity (female young/year/female)	0.143

We considered a possible underestimation of the 10% of pregnancies respect to non-pregnant females in the luteal phase.

Mean ovulation rate was 1.74±0.78 corpora lutea/female (n = 24), and all pregnant females had one embryo or fetus (1.00±0.00; n = 24). Poeppig’s woolly monkey females presented a fertilization rate of 54.3% and a mean ovum or embryo mortality of 0.83±0.70 (33.56±28.3%) per pregnancy. The fetal sex ratio for 24 pregnancies was 12 males to 12 females.

**Figure 4:**
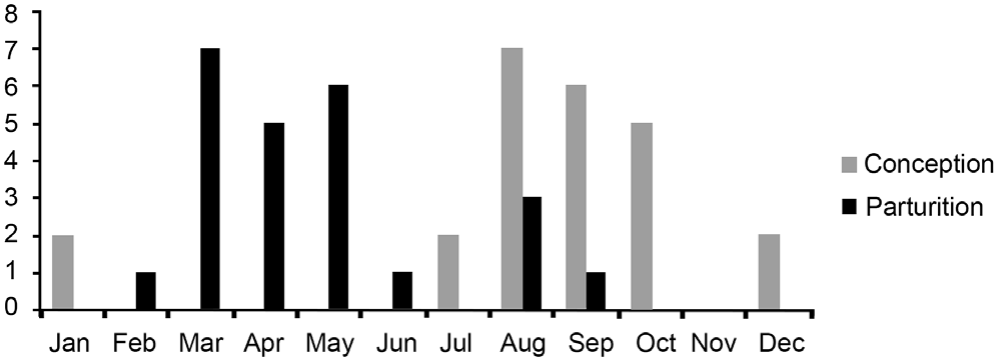
Estimated conceptions and parturitions for female Poeppigi’s woolly monkeys *Lagothrix poeppigii* (n = 24) on the Yavarí-Mirí River.

Estimated parturitions were not randomly distributed through the year (n = 24, r = 0.6355, P<0.001), occurring between March and August, whilst conceptions occurred between July and January (Figure 4). We estimate a pregnancy rate of 29–33% and a yearly reproductive production of 0.48–0.54 young per pregnant female, resulting in an interbirth interval of 22.3 to 25.2 months.

## Discussion

The rate of reproduction for woolly monkeys is considerably lower than that of other frequently hunted mammals in the Amazon region [41], [45], [46], making them more vulnerable to overhunting [47]. Woolly monkeys are being harvested on a wide scale, generally unsustainably, and this is likely to increase with the increase in oil exploitation that is predicted in many parts of Amazonia [48]–[50]. One of the major problems in the assessment of a primate population’s vulnerability to extinction is poor knowledge of its reproductive biology [51]. The widely-used Production Model [8] has been applied with minimal reproductive data for *Lagothrix*, and whilst Levi et al. [11] have developed spatial models that more accurately predict patterns of local extinction, these have also been applied with the same age at first reproduction, fecundity, and maximum longevity data used by the Production Model [8].

Reproductive rates are not a fixed constant, and can change over time and in response to local ecology. Whilst any estimate of these parameters can only be an attempt to approximate reproductive rates for a given time period and study area, our estimates use data collected over several years, which should control for some variation between years. The interbirth interval on the Yavarí-Mirí was similar to that of captive-born females (25 months [33]), and close to the widely-used estimates of Robinson and Janson [29] despite their lack of data. However, intervals for wild *Lagothrix lugens* in the Macarena Ecological Investigations Center, Colombia are longer (36.7 months, n = 13 [35]). This could reflect that these populations were not heavily hunted, whilst those on the Yavarí-Mirí were from a hunted population that may not be limited by density dependent factors. Furthermore, if animals on the Yavari-Mirí were effectively being taken from the ‘sink’ area of a source-sink system [52], groups could contain a larger proportion of newly dispersed young females with a greater chance of being pregnant rather than carrying infants.

The practical use of the Production Model [8] has been criticised for using r_max_ estimated from captive reproductive rates instead of actual population growth rates, which is said to lead to the overestimation of production, since actual population growth rates are likely to be significantly lower due to density dependence [51], [53]. Since reproductive rates at our hunted site are higher than rates recorded at sites with lower hunting pressure [35] and are more comparable with captive rates [33], our results support the use of r_max_ derived from captive populations, contra to Milner-Gulland and Akçakaya [51] and Milner-Gulland and Rowcliffe [53]. Thus the figure of 0.14 for r_max_ originally used by Robinson and Redford [8] and other sustainability models (Table 1) is probably more appropriate for *Lagothrix poeppigii* than the more recent calculation of 0.16 for captive *Lagothrix* sp. [6]. However it is clear that there is room for further refinements. For species like woolly monkeys that have proven difficult to breed in captivity [33], [54], and where management decisions may affect birth rates, it is not clear whether captive conditions will lead to higher values for *r_max_* due to abundant food, or lower values due to other factors. There is a difference between inter-birth intervals of captive wild-caught individuals and those of captive-born females [33], and also between inter-birth intervals according to infant survival [33]. These factors need consideration.

The Production Model [8] has been criticised for not taking mortality into account when calculating *r_max_* [51], [53], [55]. The interbirth interval is strongly affected by the survival of the last preceding offspring; in captive *Lagothrix* the median interbirth interval for females whose infants died was 13.3 months in contrast to 24.4 months when infants survived [33]. Slade et al. [55] provide alternatives to the Production Model that incorporate estimates of mortality, and future models, including spatial models, might similarly include measures of mortality estimated through observational fieldwork (e.g. [35]). Furthermore, infant mortality rates might vary between hunted and non-hunted populations; either though lower resource availability in non-hunted sites, or conceivably through more frequent changes in social groups in hunted sites.

Different species have physiological characteristics that determine their pattern of reproduction, but these will be modified in response to environment [56], [57]. Nutrition is linked to environmental and climatic variables, and is the main factor responsible for the seasonal reproductive pattern of non-human primates [58]. At our study site, the Poeppig’s woolly monkey appears to be an opportunist seasonal breeder capable of breeding year-around when sufficient food is available, as with the species at other study sites [35], [59]–[61]. None-the-less, seasonality in births, such as we found on the Yavarí-Mirí, might restrict potential population growth rates, perhaps restricting the lower limit of the birth interval to around 24 months to coincide with annual peaks in availability of food – a limitation that captive populations are unlikely to have. Mooney & Lee [33] observed that parturitions of the captive Poeppig’s woolly monkey were spread throughout the year, with no marked seasonality, probably due to the food supply in captive conditions. Furthermore, other woolly monkey taxa living in forests of differing seasonality, such as those in the southern extremes of Amazonia, may conceivably have differing reproductive rates, as found in callitrichids [62].

In our study, hunting registers on the Yavari-Mirí show that primates are the most hunted group and that *Lagothrix poeppigii* is one of most important prey for local people, as *Lagothrix* spp. are for indigenous and other groups throughout Amazonia [10], [63], [64], [65]. In other areas the species is also subject to non-subsistance hunting [48]–[50]. Understanding the population dynamics and the affects of hunting are key to primate conservation, but sustainability and extinction models, whilst gaining in sophistication, are limited by the reproductive parameters that they utilize, often relying on roughly estimated reproductive and life history parameters. Given the likely variation between sites and species of *Lagothrix*, and the likely use of reproductive parameters in future models of the sustainability of the widespread hunting on this genus, collecting data on these parameters, and on behavioural factors that might influence them, is vital. These data should be collected from the site being modelled whenever possible, or from sites of similar hunting pressure and seasonality for the species under study. Newer spatial models for extinction and sustainability (e.g. [11]) are subject to the same limitations of availability and accuracy of reproductive parameters and modellers might consider validating the use of captive reproductive rates by comparing with the rates in hunted populations. However, spatial models appear less sensitive to variation in reproductive parameters, constituting a further advantage to their use over the Production Model to those highlighted by Levi et al. [11]. In light of the widespread revisions in primate taxonomy, increasing sophistication of modelling, and the elevating risk of hunting to primate populations globally, these recommendations are applicable to a wide range of hunted primates in the New and Old World.

The methods we use to determine reproductive productivity are applicable to other primates, and indeed mammals. They are low-cost and simple. The only requirement is to determine the pregnancy of adult females. Although we include some ovarian here, it is not used to determine pregnancy rates. The difficulty in the methodology lies in the sample collection. Our sample collection was based on the collaboration and participation of local subsistence hunters, and assures that no animal will be killed other than those harvested as part of local hunter’s normal activities. If such methods are used, hunters need to be trained to remove all the abdominal and pelvic organs completely to avoid damage to the material. Because of the required sample sizes and the nature of the collection, in our case, it took seven years to collect enough samples. No animals should be killed specifically for the research and hunters must not be paid for the sample collection.
