# Correction: Refining Reproductive Parameters for Modelling Sustainability and Extinction in Hunted Primate Populations in the Amazon

**DOI:** 10.1371/journal.pone.0131030

**Published:** 2015-06-15

**Authors:** Mark Bowler, Matt Anderson, Daniel Montes, Pedro Pérez, Pedro Mayor

There is an error in the first equation in the Introduction. The correct equation is:

$$1=-e^{r\text{max}}+be^{-r\text{max}(a)}-be^{-r\text{max}(w+1)}$$

There is an error in the first sentence of the fifth paragraph of the Introduction. The correct sentences are: For woolly monkeys, Robinson & Redford [8] use a value for *r*_*max*_ of 0.14 calculated using the age at first (5 years) and last (20 years) reproduction and a birth rate 0.29 births per year in a captive population not restricted by density dependent factors. Our birthrate of around 0.5 births per year, in a hunted wild population, gives an *r*_*max*_ of 0.20. This 43% increase in rmax translates into a 43% increase in the potentially sustainable harvest.
